# Selective Transmission of R5 HIV-1 over X4 HIV-1 at the Dendritic Cell–T Cell Infectious Synapse Is Determined by the T Cell Activation State

**DOI:** 10.1371/journal.ppat.1000279

**Published:** 2009-01-30

**Authors:** Takuya Yamamoto, Yasuko Tsunetsugu-Yokota, Yu-ya Mitsuki, Fuminori Mizukoshi, Takatsugu Tsuchiya, Kazutaka Terahara, Yoshio Inagaki, Naoki Yamamoto, Kazuo Kobayashi, Jun-ichiro Inoue

**Affiliations:** 1 Department of Immunology, National Institute of Infectious Diseases, Shinjuku-ku, Tokyo, Japan; 2 Division of Cellular and Molecular Biology, Department of Cancer Biology, Institute of Medical Science, University of Tokyo, Minato-ku, Tokyo, Japan; 3 Department of Molecular Virology, Bio-Response, Tokyo Medical and Dental University, Bunkyo-ku, Tokyo, Japan; 4 AIDS Research Center, National Institute of Infectious Diseases, Shinjuku-ku, Tokyo, Japan; Harvard Medical School, United States of America

## Abstract

Dendritic cells (DCs) are essential antigen-presenting cells for the induction of T cell immunity against HIV. On the other hand, due to the susceptibility of DCs to HIV infection, virus replication is strongly enhanced in DC–T cell interaction via an immunological synapse formed during the antigen presentation process. When HIV-1 is isolated from individuals newly infected with the mixture of R5 and X4 variants, R5 is predominant, irrespective of the route of infection. Because the early massive HIV-1 replication occurs in activated T cells and such T-cell activation is induced by antigen presentation, we postulated that the selective expansion of R5 may largely occur at the level of DC–T cell interaction. Thus, the immunological synapse serves as an infectious synapse through which the virus can be disseminated *in vivo*. We used fluorescent recombinant X4 and R5 HIV-1 consisting of a common HIV-1 genome structure with distinct envelopes, which allowed us to discriminate the HIV-1 transmitted from DCs infected with the two virus mixtures to antigen-specific CD4^+^ T cells by flow cytometry. We clearly show that the selective expansion of R5 over X4 HIV-1 did occur, which was determined at an early entry step by the activation status of the CD4^+^ T cells receiving virus from DCs, but not by virus entry efficiency or productivity in DCs. Our results imply a promising strategy for the efficient control of HIV infection.

## Author Summary

The cellular tropism of HIV-1 is determined by the binding of HIV-1 envelope to chemokine coreceptors, CCR5 or CXCR4, in addition to a major entry receptor, CD4. The mystery still now is that despite the mixed infection of CCR5-utilizing (R5) and CXCR4-utilizing (X4) HIV-1 in many AIDS patients, R5 is predominantly isolated from newly infected individuals whatever the mode of infection. Because the early massive HIV-1 replication occurs in activated T cells and such T-cell activation is induced initially by antigen-presenting DCs, we postulated that the selective expansion of R5 may largely occur at the level of antigen-dependent DC–T cell interaction, called immunological synapse. Thus, the immunological synapse serves as an infectious synapse through which the virus can be rapidly disseminated *in vivo*. In this study, we prepared X4 and R5 HIV-1 expressing red or green fluorescence and showed that the selective expansion of R5 over X4 did occur, depending on the activation status of CD4^+^ T cells receiving virus from DCs, but not by virus entry efficiency or productivity in DCs.

## Introduction

HIV-1 infects T cells and monocyte lineage cells, including macrophages and dendritic cells (DCs), through CD4, the primary receptor for entry. The cellular tropism of HIV-1, i.e., macrophage (M)-tropic or T-cell line (T)-tropic, is determined by chemokine receptors. Depending on whether they mainly use the CCR5 or CXCR4 entry coreceptors, primary isolates are defined as R5 for M-tropic and X4 for T-tropic variants, respectively [1]. Despite the fact that the HIV-1 present in infected individuals frequently comprises the mixture of R5 and X4, virus isolated from individuals newly infected through sexual, parenteral, or mother-to-child transmission is also predominantly R5 [2],[3],[4]. During the clinical course of disease progression, the phenotype of the virus may evolve from R5 to X4 or to R5/X4-dual tropic [5],[6],[7], and X4 virus has been shown to be associated with a decline in CD4^+^ T cell counts and the onset of clinical symptoms of AIDS [8]. However, R5 and X4 viruses are equally cytopathic [9], and R5 virus isolated from patients with late-stage disease are similarly pathogenic to X4 *in vitro* [10]. These findings suggest that an yet-unknown selective mechanism that favors R5 virus exists during transmission and/or the early phases of infection in the host (review in [11]).

DCs are important antigen-presenting cells that initiate an immune response by activating naïve and memory CD4^+^ T cells [12]. Although it is known that DCs are susceptible to HIV-1 infection, virus productivity from DCs and R5/X4 preferences for DCs vary (see review in [13]). This could be attributed in large part to the heterogeneous nature of DC sources, maturation levels, proliferative capacities, methods for isolation, and culture conditions. Importantly, all DC subsets express CD4 and varying levels of CXCR4 and CCR5.

Because of the low frequency of DCs *in vivo,* blood monocytes are often utilized as representative myeloid DCs for the study of HIV-1 infection. We showed earlier that although monocyte-derived DCs (MDDCs) generated *in vitro* are susceptible to X4 and R5 HIV-1 infection, R5-infected DCs are poorly productive compared with R5-infected macrophages of the same monocyte origin [14]. Nevertheless, HIV-infected MDDCs efficiently transmit virus to autologous CD4^+^ T cells [15],[16],[17], by close contact between MDDCs and CD4^+^ T cells. Thus, when HIV-infected DCs present antigens to CD4^+^ T cells in lymphoid organs, an immunological synapse is formed and a T cell–activation program proceeds, which allows virus transmitted from DCs to replicate in activated CD4^+^ T cells. This interaction is called an infectious synapse [18],[19]. Thus, efficient HIV transmission from DCs to CD4^+^ T cells through infectious synapses may play a central role not only for the massive expansion of HIV following initial infection, but also for generating latent infection in HIV-specific memory CD4^+^ T cells [13].

The expression level of CXCR4 does not appear to be a crucial factor of X4 replication, because most circulating hematopoietic cells, including CD4^+^ T cells and DCs [20], or submucosal lymphocytes [21] express CXCR4 albeit at various levels. Although the abundant CCR5 expression in activated/memory CD4^+^ T cells in submucosa may explain the preferential sexual transmission of R5 HIV-1, there are many more CXCR4^+^ CD4^+^ T cells than there are CCR5^+^ CD4^+^ T cells in the blood [11],[22]. Furthermore, MDDCs, and macrophages from the same individual express similarly low levels of CCR5 [23] despite large differences in R5 virus productivity. Cavrois et al. recently analyzed the fusion activity of labeled virion with DC membranes and showed that the fusion efficiency of R5 declined as DCs matured and CCR5 expression decreased, and that X4 fusion efficiency did not change with maturation [24]. On the other hand, Pion et al. showed that fusion of X4 with immature DCs was markedly inefficient compared with that of R5, and that this inefficiency was not complemented by ectopic expression of CXCR4 [25]. They hypothesized that an as-yet unknown env-specific block early in the virus infection cycle occurs in DCs, which is not due solely to surface expression level of chemokine receptors.

The state of T-cell activation determines the level of HIV-1 replication. HIV-1 replication in resting primary CD4^+^ T cells is inefficient at every level after entry: reverse transcription, nuclear import, integration, and transcription [26],[27]. Interestingly, a significant replicative advantage of R5 over X4 HIV-1 in some CD4^+^ T cell clones is reported and X4-dependent restriction of HIV replication is rescued by T cell receptor (TcR) stimulation [28]. In TcR-stimulated CD4^+^ T cells, R5, but not X4, HIV-1 efficiently replicates in the absence of MEK/ERK signaling, whereas nuclear import of X4 HIV-1 is dependent on the MEK/ERK pathway [29]. Recently, Cicala et al. showed that R5 ENV up-regulates the expression of genes belonging to MAP kinase pathways and genes regulating the cell cycle to a greater extent than X4 ENV [30]. Stronger modulation of transcription by R5 than by X4 viruses in CD4^+^ T cells was also reported [31]. These results suggest that R5 HIV-1 has an advantage in establishing the infection cycle in CD4^+^ T cells. The question is how this mechanism contributes to the selective expansion of R5 virus early in HIV-1 infection.

To study the preference of R5 or X4 HIV-1 transmission during DC-T cell interaction, we produced highly replication-competent, fluorescent viruses of X4 and R5 type. We analyzed the HIV-1 life cycle in MDDCs and CD4^+^ T cells, before and after coculture, by quantitative PCR (qPCR) and FACS. Although the infection process progressed at an equal rate in MDDCs infected with either R5 or X4 virus, R5 virus predominantly replicated in CD4^+^ T cells which are activated by antigen-presenting HIV-infected MDDCs.

## Results

### HIV-1 expressing EGFP or DsRed is replication competent in PHA-activated PBMCs

**Figure 1:**
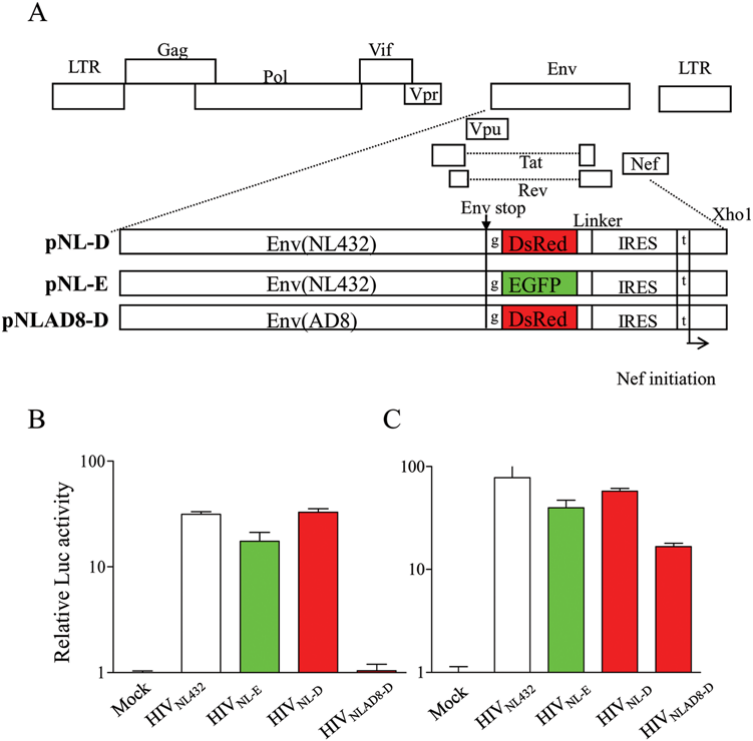
Genomic structure and co-receptor usage of recombinant HIV-1 encoding EGFP or DsRed. (A) The structure of provirus DNA encoding *EGFP* or *DsRed* designated as pNL-E and pNL-D for X4 HIV-1 and pNLAD8-D for R5 HIV-1. EGFP or DsRed is not expressed as a fusion protein of Env because of one base insertion after the Env stop codon. Nef is also independently expressed under the control of IRES. To confirm the coreceptor usage of these fluorescent HIV-1, 1G5 (B) cells, 1G5/CCR5 (C) cells were infected with HIV-1_NL432_ (parent strain), HIV-1_NL-E_, HIV-1_NL-D_, or HIV-1_NLAD8-D_. After 48 h post-infection, cell lysates were prepared and the Luc assay was performed. The data represents the averages ±SD of three independent experiments.

We generated fluorescent X4 (HIV-1_NL-D_) and R5 (HIV-1_NLAD8-D_) viruses. The structure of these provirus clones and HIV-1_NL-E_ [32] was depicted in Fig. 1A. The co-receptor usage of these viruses was determined by 1G5 or1G5/CCR5 cells, which contain a LTR-driven luciferase gene [33]. As shown in Fig. 1B, both 1G5 and 1G5/CCR5 are infected with X4 type virus (HIV-1_NL-E_ and HIV-1_NL-D_), whereas 1G5/CCR5, but not 1G5, is infected with R5 HIV-1 (HIV-1_NLAD8-D_), indicating the expected coreceptor usage.

**Figure 2:**
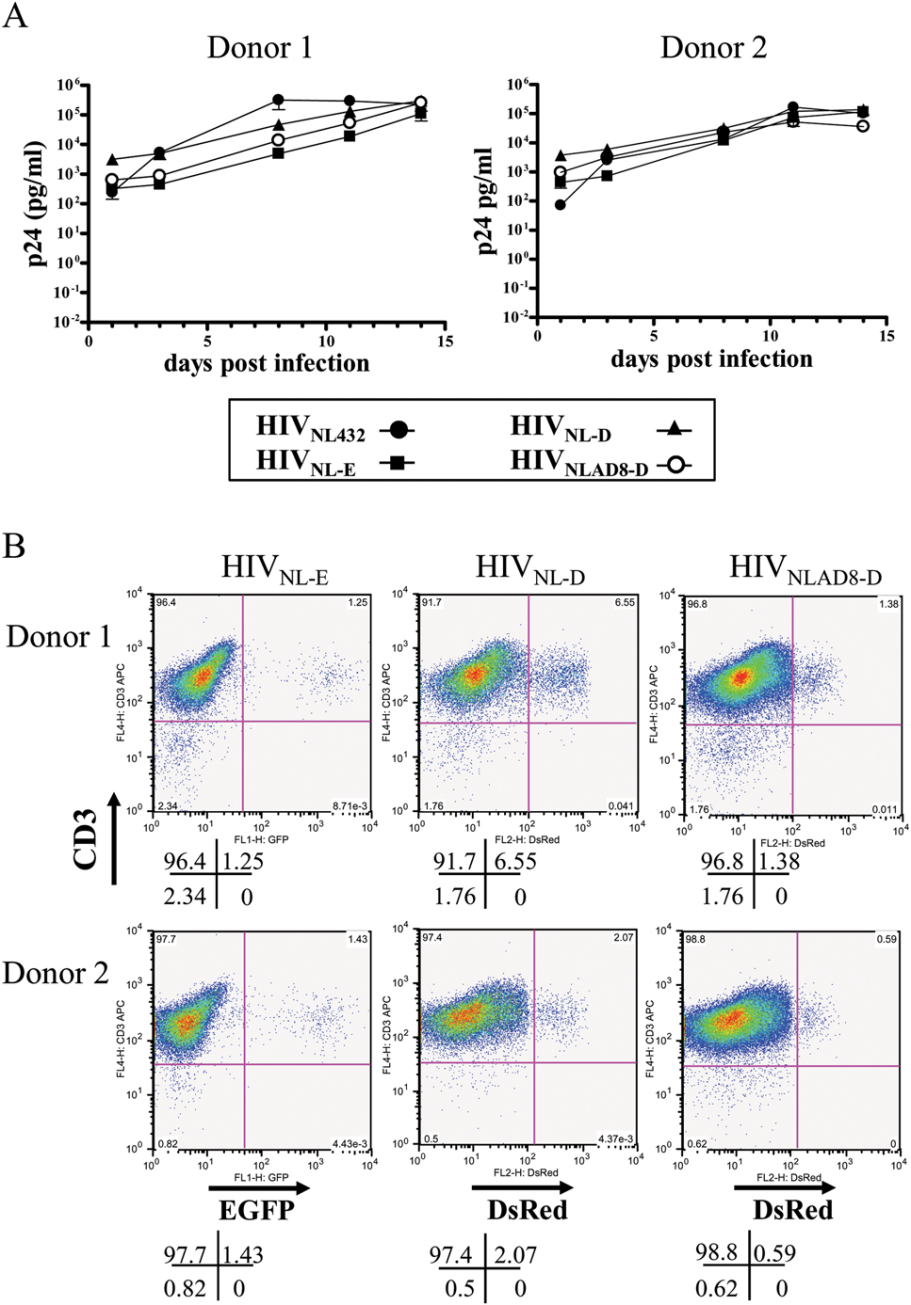
Replication of recombinant HIV-1 encoding EGFP or DsRed. (A) Concentration of X4 HIV-1 (HIV_NL-432_), X4 HIV-1 expressing EGFP (HIV_NL-E_), X4 HIV-1 expressing DsRed (HIV_NL-D_), or R5HIV-1 expressing DsRed (HIV_NLAD8-D_) in PHA-stimulated PBMCs of two donors. Virus production was monitored by in-house p24 antigen ELISA. (B) FACS analysis of HIV-1-infected T cells expressing EGFP or DsRed at 7 dpi.

By combining X4 HIV-1_NL-E_ and R5 HIV-1_NLAD8-D_, it became easy to monitor their replication in individual cells using FACS. These viruses were prepared by transfecting proviral DNA into 293T cells. We infected PBMCs stimulated with phytohemagglutinin (PHA blasts) from two donors with the same p24 Gag amount of the prototype virus HIV-1_NL432_ or with fluorescent viruses HIV-1_NL-E_, HIV-1_NL-D_, or HIV-1_NLAD8-D_ and then monitored the kinetics of HIV-1 replication (Fig. 2A). Because *nef* is not deleted in these constructs, the infectivity of the fluorescent viruses is preserved and comparable to that of wild-type virus. When cells were analyzed by FACS at 7 d post-infection (dpi), we were able to clearly detect EGFP- or DsRed-positive CD3^+^ T cell populations (Fig. 2B). Therefore, these fluorescent viruses are useful tools with which to identify HIV-infected cell populations.

### HIV-1 transmitted from infected MDDCs to CD4^+^ T cells during antigen presentation is predominantly the R5 variant

**Figure 3:**
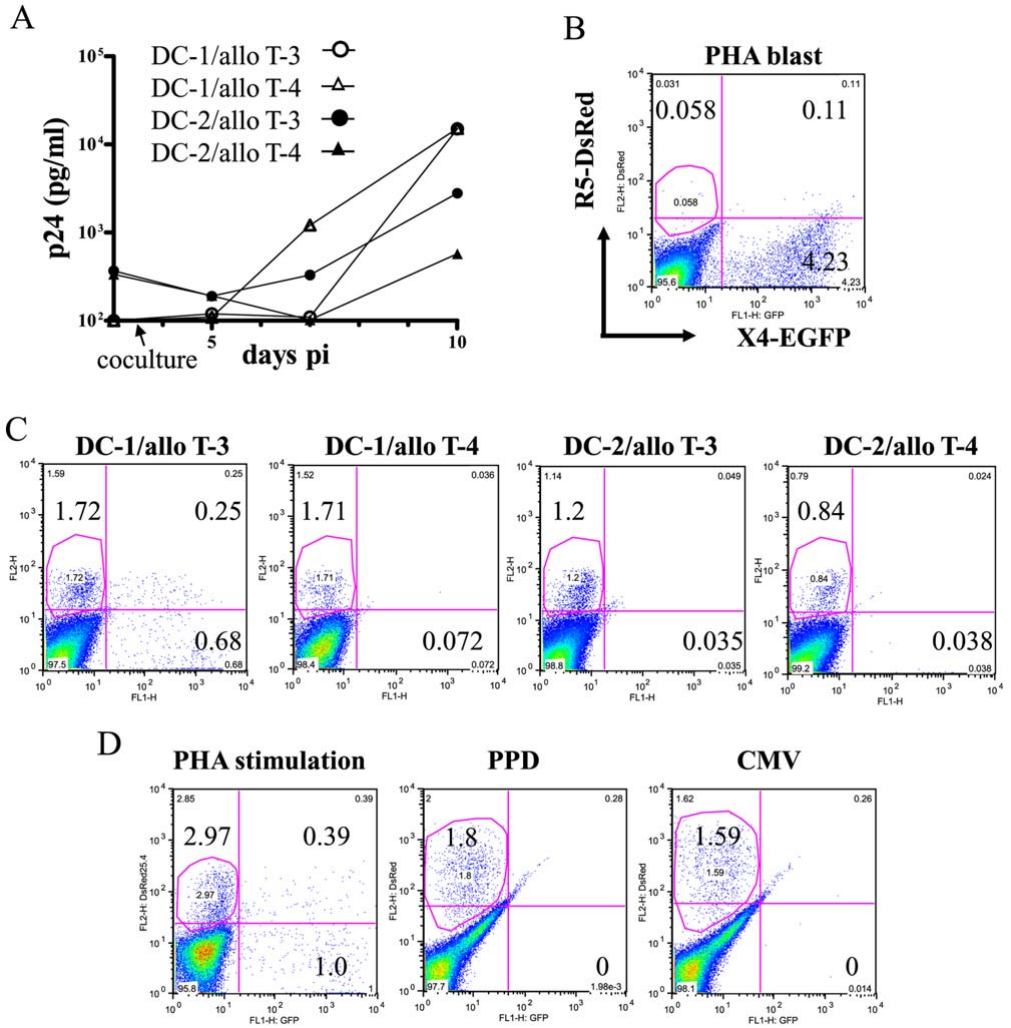
Transmission of HIV-1 from infected MDDCs to CD4^+^ T cells. (A) Virus production as measured by in-house p24 antigen ELISA in CD4^+^ T cells cocultured with MDDCs infected with the same amount of HIV_NL-E_ and HIV_NLAD8-D_ Culture supernatants were harvested every 3–4 d. (B) FACS analysis of PHA blast T cells which were directly infected with the mixture of R5 and X4 HIV-1 at 7 dpi. (C) allogeneic CD4^+^ T cells cocultured with HIV-infected MDDCs at 10 dpi. Live (PI^−^) and CD3^+^ T cells were gated. (D) FACS analysis of autologous CD4^+^ T cells cocultured with infected MDDCs in the presence of PHA (left), PPD antigen (middle) or CMV-infected cell lysate (right). Live (PI^−^) and CD3^+^ T cells were gated.

Next, we determined which HIV-1 variant preferentially replicates in CD4^+^ T cells that have been activated by interacting with HIV-infected immature MDDCs. We mixed an equal amount of p24-measured HIV-1_NL-E_ and HIV-1_NLAD8-D_, and infected MDDCs. We then cocultured the infected MDDCs with allogeneic CD4^+^ T cells. As shown in Fig. 3A, HIV-1 replication was detectable at 7 dpi in the culture supernatant of the MDDC–T cell coculture of DCs (DC-1 or DC-2) and CD4^+^ T cells (allo T-3 or allo T-4). As reported previously, MDDCs themselves produce little if any virus during cultivation [14], and we were unable to detect EGFP^+^ or DsRed^+^ MDDCs at 7 dpi (data not shown). When day 3 PHA blasts were directly infected with the virus mixture, cells infected with X4 viruses expressing EGFP (HIV-1_NL-E_) predominated at 7 dpi (Fig. 3B). This may be due to the reduced expression of CCR5 in day 3 PHA blasts [22]. In contrast, in most of the combinations of MDDCs and allogeneic CD4^+^ T cells (more than 10), cells infected with R5 virus expressing DsRed (HIV-1_NLAD8-D_) were the predominant population, producing virus at 10 dpi (Fig. 3C). The representative results of two MDDC donors (DC-1 and DC-2) cocultured with allogeneic T cells from two donors (T-3 and T-4) were shown here. Of note, a substantial replication of X4 virus was detected only in DC-1/allo T-3 combination, indicating that the activation of this donor's CD4^+^ T cells (T-3) by allogeneic DC (donor-1) is exceptionally powerful.

We also examined the replicability of R5 and X4 virus under physiological conditions in which DC–T cell interactions occur during antigen-specific immune responses. As shown in Fig. 3D, R5 virus replicated predominantly in PPD- and CMV-reactive CD4^+^ T cells at 9 dpi (middle and right). In PHA- stimulated T cells, however, both R5 and X4 virus were able to replicate at 7 dpi (left), which is quite similar situation to DC-1/allo T-3 combination (Fig. 3C). We obtained consistent results with cells from several donors during both allogeneic and antigen-specific interactions between MDDCs and CD4^+^ T cells. Our results suggest that R5 virus has an advantage over X4 virus during transmission from MDDCs to CD4^+^ T cells.

### Similar Infectivity of X4 and R5 HIV-1 in MDDCs

**Figure 4:**
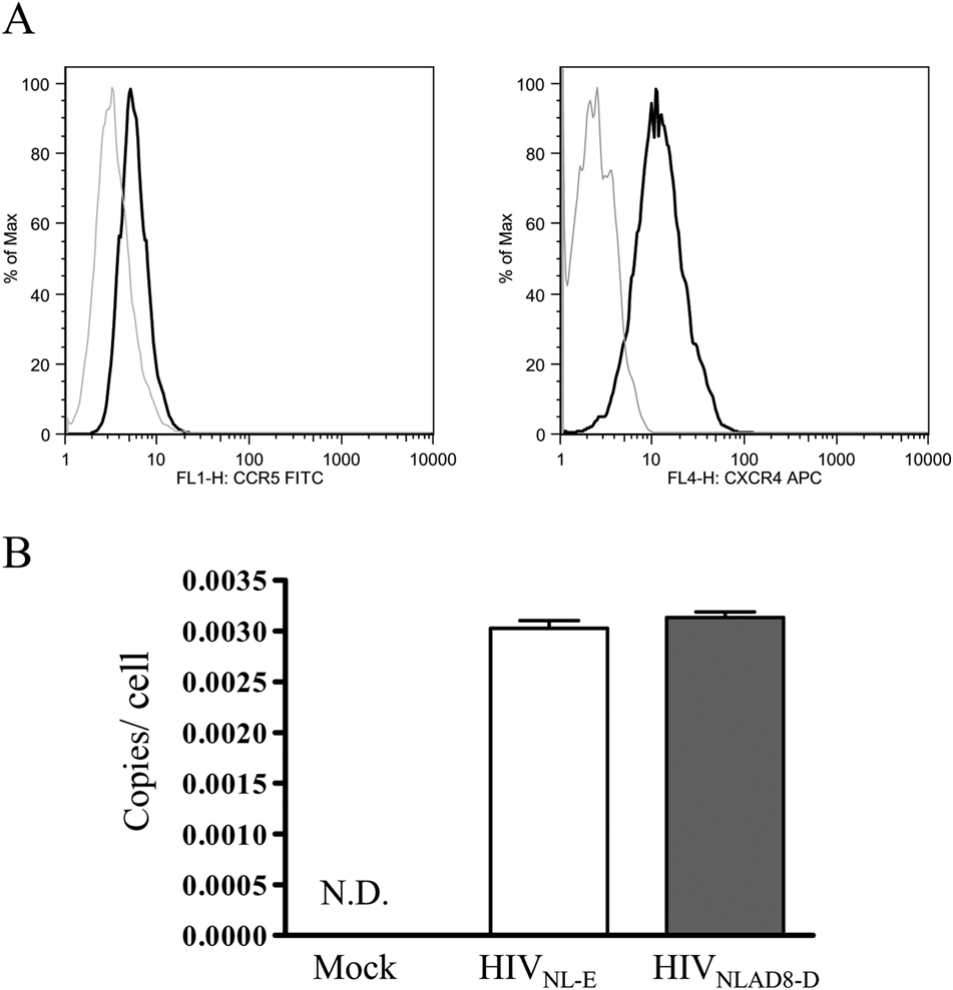
Infectivity of HIV-1 in MDDCs. (A) Surface expression of CCR5 and CXCR4 in MDDCs. MDDCs were stained with anti-CCR5 (left) and anti-CXCR4 mAb (right), or with isotype control mAbs (dotted line). The reproducible representative of the FACS profiles of several individuals is depicted. (B) Quantitative PCR analysis of R5 (HIV_NLAD8-D_) and X4 (HIV_NL-E_) HIV-1-infected MDDCs. Data represent the average ±SD of three independent experiments.

There is some controversy regarding the difference between X4 and R5 virus susceptibility among DC subsets. Some reports indicate that immature MDDCs are more susceptible to R5 virus than to X4 [16],[34],[35], which may partly explain predominant R5 transmission. Therefore, we felt that it was necessary to determine the efficiency of infection of X4 and R5 in MDDCs. First, we checked the level of coreceptor expression in immature MDDCs. The representative results of several individuals are shown in Fig. 4A. The lower expression level of CCR5 compared with CXCR4 (Fig. 3A) in immature MDDCs is quite consistent with the pattern reported by Cavrois et al. [24], who utilized exactly the same protocol for MDDC preparation.

To analyze early steps after HIV-1 entry, we infected MDDCs with the same p24-measured amount of either HIV-1_NL-E_ or HIV-1_NLAD8-D_ and prepared cell lysates at 8 h post-infection (hpi). We measured the amount of distinct forms of proviral DNA (*R-U5* and *U5-gag*) by qPCR as previously described [36]. The amount of these DNA forms was normalized to that of β-globin. Unfortunately, the copy number of *R-U5*, the earliest reverse transcription product in MDDCs, was too low and varied too much among individuals to allow us to evaluate the differences in entry step of X4 and R5. However, similar amounts of the late reverse transcription product *U5-gag* were consistently detected in X4- or R5-infected MDDCs. A representative result is shown in Fig. 4B. We also tested the infectivity of these viruses in MDDCs by utilizing HIV-1 in which the gene encoding *Renilla* luciferase (hRluc) is inserted in replacement of DsRed or EGFP gene. The hRluc activities of R5 and X4 viruses did not differ in infected MDDCs at 3 dpi (data not shown). Thus, our results suggest that selective transmission of R5 over X4 HIV-1 from DCs to T cells is not due to differences in early entry, reverse transcription, integration, or transcription in MDDCs.

### Selective transmission of R5 over X4 HIV-1 through an infectious synapse depends on the T cell activation state

**Figure 5:**
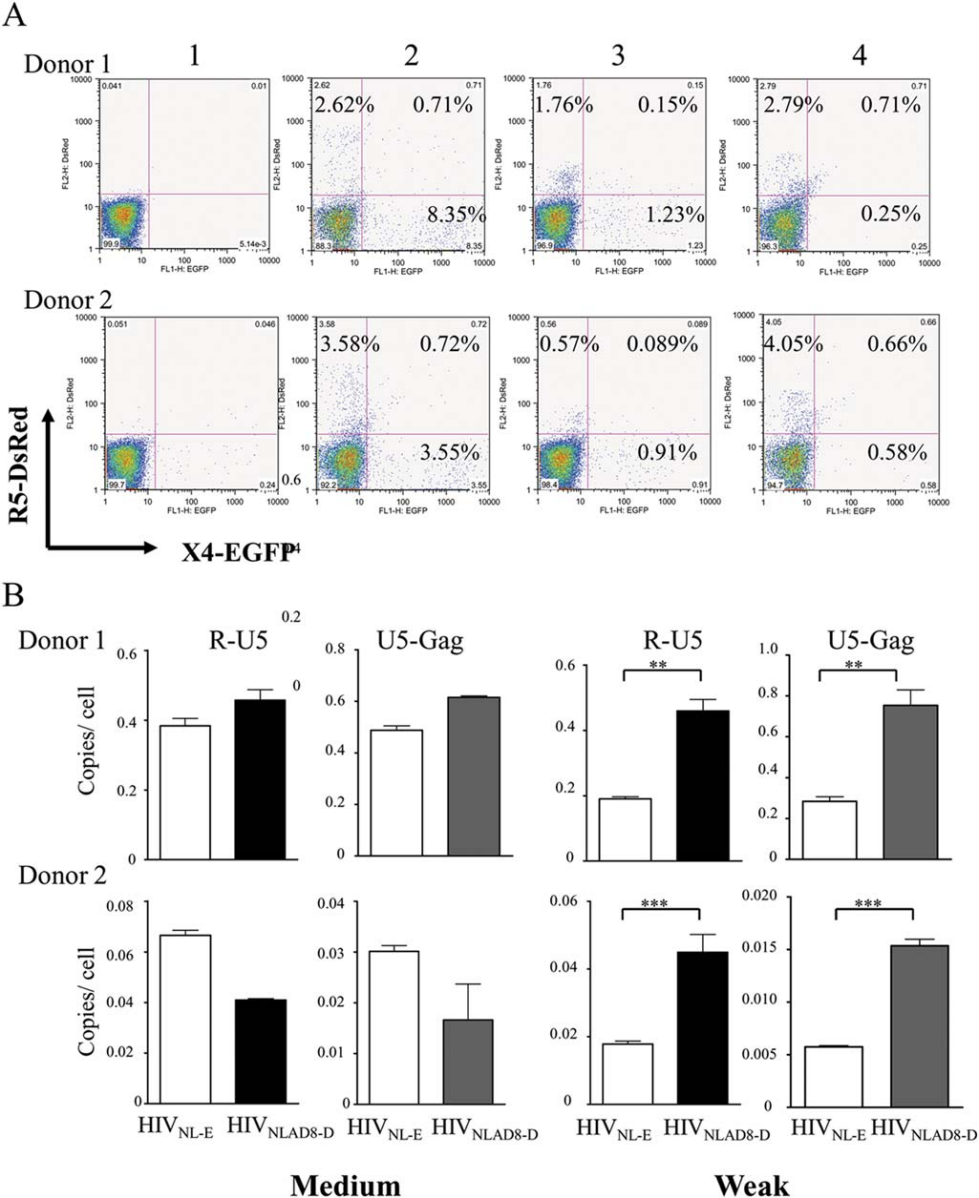
FACS-based analysis of dual infection in primary CD4^+^ T cells. (A) FACS analysis of primary CD4^+^ T cells (1) unstimulated, (2) stimulated with 5 µg/ml anti-CD3 and 10 µg/ml anti-CD28 for 24 h (strong activation), (3) stimulated with the same concentrations of anti-CD3 and anti-CD28 for 2 h (medium activation), or (4) stimulated with 10-fold lower concentrations of anti-CD3 and anti-CD28 for 2 h (weak activation). Cells were then infected with equal amounts of X4 and R5 HIV-1 and analyzed at 5 dpi. (B) Quantitative PCR analysis of CD4^+^T cells separately infected with either R5 or X4 HIV-1. R-U5 and U5-Gag was analyzed by qPCR in two donors. The amount of HIV-1–specific DNA per cell was normalized to β-globin gene expression. The data represents the average ±SD of three independent experiments. **, *P*<0.005; ***, *P*<0.0005.

Suppose R5 and X4 viruses infect MDDCs and are transmitted to CD4^+^ T cells with similar efficiency, it could be that selective replication of R5 virus in DC–T cell coculture depends on the state of T cell activation. To determine whether this is so, we controlled the activation state of CD4^+^ T cells by varying TcR-stimulation conditions, and then we analyzed the infectivity of HIV-1_NL-E_ and HIV-1_NLAD8-D_ in these cells (Fig. 5A). Prior to infection with HIV-1, autologous CD4^+^ T cells were (1) unstimulated, (2) stimulated with 5 µg/ml anti-CD3 and 10 µg/ml anti-CD28 for 24 h (strong activation), (3) stimulated with the same concentrations of anti-CD3 and anti-CD28 for 2 h (medium activation), or (4) stimulated with 10-fold lower concentrations of anti-CD3 and anti-CD28 for 2 h (weak activation). These CD4^+^ T cells were infected with the virus mixture and analyzed at 5 dpi. Fig. 5 shows the reproducible representative results in cells from two of the six donors. Notably, in the weakly activated CD4^+^ T cells, only R5 HIV-1 replicated (Fig. 5A-4), whereas both X4 and R5 virus replicated in different cells following medium activation (Fig. 5A-3), and cells that were doubly infected with both X4 and R5 were detected after strong activation condition (Fig. 5A-2). Using the same weak and medium activation conditions, we quantified the early *R-U5* and *U5-gag* forms of HIV-1 reverse transcription products in CD4^+^ T cells in these donors at 8 hpi. The amount of *R-U5 and U5-gag* DNA did not differ significantly between X4 HIV-1- and R5 HIV-1-infected CD4^+^ T cells following medium activation (Fig. 5B, left, blank and filled column, respectively). Surprisingly, however, after weak activation, the amount of proviral DNA was dramatically higher in the R5 HIV-1-infected cells compared with those cells infected with X4 (Fig. 5B, right) (**P<0.005 and ***P<0.0005, in Donor 1 and 2, respectively). These results suggest that the activation state of CD4^+^ T cells is a key factor in determining selective R5 HIV transmission and virus expansion during DC–T cell interactions.

**Figure 6:**
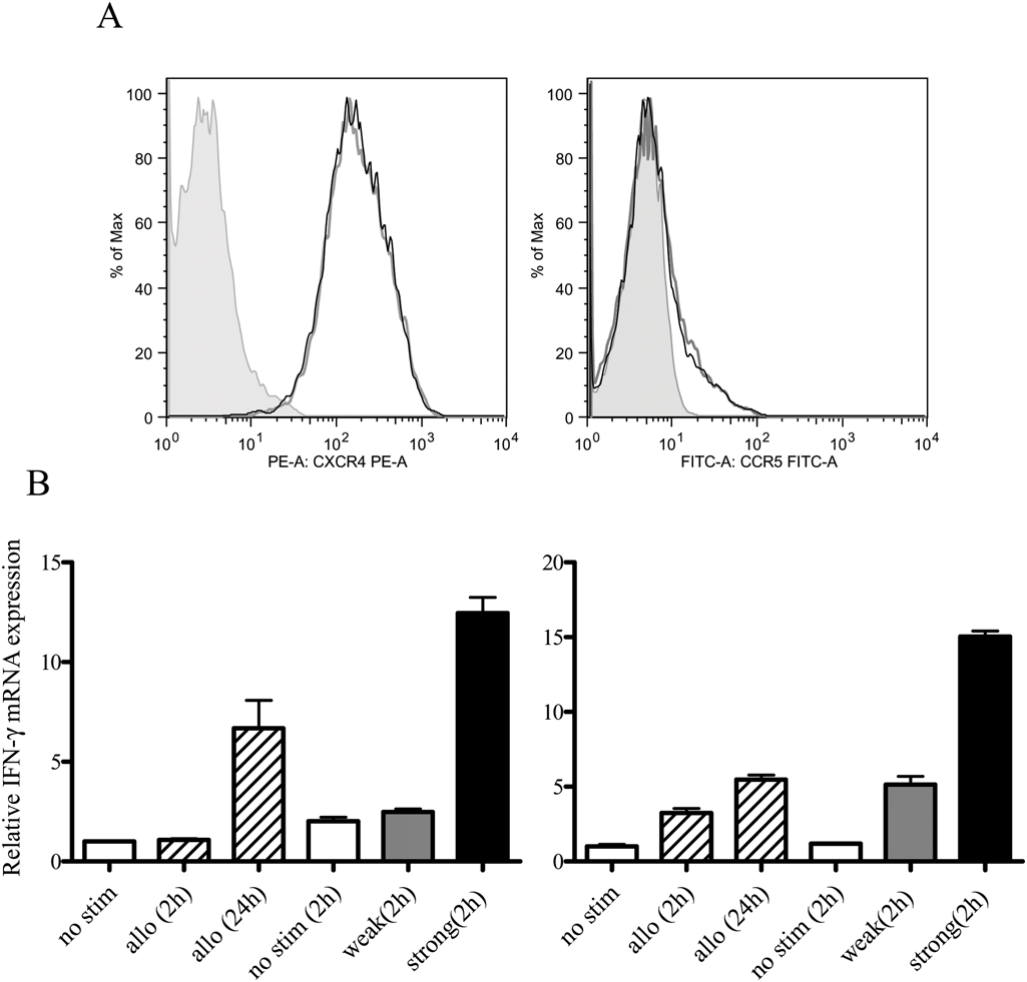
Analysis of CD4^+^ T cell activation. (A) Primary CD4^+^ T cells were stimulated with anti-CD3 (5 µg/ml)+anti-CD28 (10 µg/ml) (black bold line), anti-CD3 (0.5 µg/ml)+anti-CD28 (1 µg/ml) (black line), or cocultured with allogeneic CD4^+^ T cells for 2 h (gray line). Isotype control mAbs were used as a negative staining control (shaded peak). (B) One-step qRT-PCR analysis of IFN-γ mRNA expression in primary CD4^+^ T cells. Primary CD4^+^ T cells were cocultured with allogeneic CD4^+^ T cells for 2 or 24 h and then stimulated with anti-CD3 (5 µg/ml)+anti-CD28 (10 µg/ml) (IL-2 strong), anti-CD3 (0.5 µg/ml)+anti CD28 (1 µg/ml) (IL-2 weak), or no TCR stimulation (IL-2) for 2 h. Total RNA was extracted and analyzed by qRT-PCR. The data was normalized to EF-1α and the relative amount of IFN-γ is shown. The data represents the average ±SD of three independent experiments.

As shown in Fig. 3, R5 was the HIV-1 variant predominantly transmitted during antigen-specific DC and CD4^+^ T cell interaction. To determine whether or not the activation state of CD4^+^ T cells stimulated by antigen-presenting MDDCs is relevant to that of CD4^+^ T cells weakly TcR stimulated with anti-CD3 and anti-CD28, we first analyzed the expression levels of CCR5 and CXCR4 on CD4^+^ T cells activated by anti-CD3 and anti-CD28 or MDDCs (Fig. 6). Primary resting CD4^+^ T cells were cocultured with allogeneic MDDCs (allo) for either 2 or 24 h. Alternatively, primary resting CD4^+^ T cells were either unstimulated, strong or weak TcR stimulated for 2 h. We did not observe a substantial difference with respect to the surface expression of HIV entry coreceptors 2 h stimulation following any of the conditions. The FACS profile of unstimulated and strongly TcR-stimulated CD4^+^ T cells is depicted in Fig. 6A. Although these cells were also analyzed for surface activation markers (CD69, CD25, and HLA-DR), no difference was observed. We, therefore, compared the mRNA expression levels of IFN-γ and IL-2, two representative markers of early TcR activation, in these cells by quantitative reverse transcription-PCR (qRT-PCR). The results in cells from two donors are shown in Fig. 6B. The expression level of IFN-γ in allo (oblique lined column) and weak (grey column) stimulation was similar at 2 h and it increased more than 10-fold in strong (black column) stimulation. After 24 h of weak stimulation, IFN-γ increased to a level equivalent to that seen after 2 h strong stimulation (data not shown), but IFN-γ expression in allo-stimulated CD4^+^ T cells did not reach the maximum level even after 24 h. In both donors, IL-2 mRNA expression was detectable only after strong stimulation for 24 h (data not shown). Thus, both X4 and R5 HIV-1 replicate well in strongly activated CD4^+^ T cells, but R5 virus is the variant capable of replicating in CD4^+^ T cells during DC-mediated antigen-specific activation, which may be more closely mimic *in vivo* situation.

## Discussion

Why HIV-1 isolated from individuals newly infected with both R5 and X4 variants should be predominantly R5, irrespective of the route of infection, is a longstanding discussion [11]. Because DCs are one of the initial targets for HIV-1 infection and a source of virus dissemination, their role in AIDS pathogenesis has also been the recent topic of much discussion [13],[19],[37]. HIV-infected DCs, albeit at low productivity, may form infectious synapses with CD4^+^ T cells during antigen-specific immune response in draining lymph nodes, and efficient HIV-1 transmission and expansion may occur in this microenvironment. The question that we sought to address was whether the predominance of R5 HIV-1 over X4 was determined at the level of DCs or CD4^+^ T cells and by what mechanism. By analyzing R5 selection during antigen-dependent MDDC–CD4^+^ T cell HIV-1 transmission, we showed that MDDCs were infected with R5 and X4 HIV-1 and produced low but similar levels of proviral DNAs. We also found that while HIV-1 transmission from MDDCs to CD4^+^ T cells was predominantly R5, transmission from MDDCs to preactivated CD4^+^ T cells *in vitro* was slightly more X4 predominant. Although the possibility that this R5 dominancy is ascribed to a unique property of HIV-1_ADA_ envelope is not formally excluded, we showed here that the selective expansion of R5 over X4 *in vivo* may be determined by the activation status of CD4^+^ T cells, but not by the efficiency of virus entry or productivity in MDDCs. The CCR5 and CXCR4 expression levels appear to contribute little to the predominance of R5 HIV-1 transmission from MDDCs to CD4^+^ T cells.

DCs are thought to be involved in the sexual transmission of HIV-1 [37]. Rescigno et al. showed that murine submucosal DCs extend their dendrites to the intestinal luminal surface [38], indicating that it may be possible for HIV to directly come into contact with and infect DCs, although how susceptible human submucosal DCs are to X4 and R5 HIV-1 infection is not known. Supporting an alternative infection hypothesis, Bomsel showed that HIV-1 entered through the epithelial barrier and was transmitted to submucosal DCs and CD4^+^ T cells by transcytosis [39]. Interestingly, Meng et al. reported that human mucosal epithelial cells express CCR5, but not CXCR4. By infecting these cells with equal amounts of R5 and X4 virus, only R5 HIV-1 variant was transmitted, which was determined based on the sequence of the gp120 *env* V3 region [40]. In contrast, Bobardt et al. recently demonstrated that genital epithelia expresses low levels of CCR5 and CXCR4 and that only limited amounts of HIV-1 were transcytosed, without no preference for R5 or X4 [41]. In a monkey model of intravaginal simian immunodeficiency virus (SIV) infection, the infected cells appeared to be DCs in the lamina propria or intraepithelium [42],[43]. However, because of the low productivity HIV-1 in DCs, it will be quite difficult to clarify the role of submucosal DCs during early HIV-1 infection in humans. What is most important, however, is not the level of HIV-1 productivity in infected submucosal DCs, but the migratory nature of these DCs to regional lymph nodes.

Resting primary CD4^+^ T cells are refractory to viral replication; the magnitude of viral replication in these cells is closely linked to their activation state [44]. The R5 HIV-1 envelope protein is known to deliver a signal that activates T cells to some extent [30], which may assist R5 HIV-1 following cell entry to accomplish reverse transcription, nuclear transport, and integration. The lack of such a signal through CXCR4 may explain why X4 virus is more strongly restricted than is R5 virus in primary CD4^+^ T cells [29]. In fact, we showed that weak activation conditions support R5 HIV-1 replication, whereas stronger activation conditions support replication of both viruses equally well (Fig. 4). When MDDCs interacted with alloantigen- or nominal antigen-specific CD4^+^ T cells through an immunological synapse, T cells received a signal from TcR first, followed by secondary signals from costimulatory adhesion molecules, resulting in early cytokine production. The intracellular environment of activated T cells under these conditions may be akin to the weak activation of T cells by TcR. The expression level of IFN-γ 2 h after T cell activation by alloantigen or by weak TcR stimulation was similarly low compared to that by strong TcR stimulation (Fig. 6B). We conclude from these results that the initial T-cell activation occurs weakly during DC–CD4^+^ T cell interactions, and that this low level of activation is enough for R5, but not for X4, to establish the viral life cycle. It is known that the retrovirus integration in a single cell is somehow limited process [45]. The CD4^+^ T cells may have come into contact with R5 and X4 HIV-1 produced by DCs with similar efficiency through the synapse, allowing the two variants to enter simultaneously into cells. However, once R5 HIV-1 is integrated in weakly activated CD4^+^ T cells, a delayed progression of X4 HIV-1 life cycle may be outcompeted by R5 HIV-1 even if the CD4^+^ T cells are later fully activated.

In summary, we visualized selective replication of the R5 HIV-1 variant following interactions between infected MDDCs and CD4^+^ T cells. We do not ascribe this to increased infectivity of R5 virus over X4 virus in MDDCs, but, rather, to the activation state of CD4^+^ T cells when they encounter a low level of these viruses at the infectious synapse. It is during antigen-specific interactions between DCs and CD4^+^ T cells that HIV-1 transmission occurs most efficiently *in vivo,* and it is during these interactions that R5 virus replicates preferentially over X4 virus.

## Methods

### Construction of plasmids

The plasmid pNL-E is a pNL432 (GenBank #M19921)-based proviral clone expressing EGFP, as described previously [32]. To create the pNL432-based proviral clone pNL-D, which expresses DsRed, a fragment of the DsRed gene, along with the pNL432 *env* region from the Hpa I digestion site to 3′ end of the *env*, was amplified by PCR, digested with Hpa I and Not I, and then ligated into the corresponding site in pNL-E. The EGFP and DsRed genes are located downstream of *env*, followed by an internal ribosome entry site (IRES) and *nef.*

To generate R5 tropic HIV-1, we constructed a proviral clone called pNLAD8-D by digesting pNL432-based pNLAD8 DNA (*env* is originated from HIV-1_ADA_ strain, kindly provided by Michael W. Cho, Department of Medicine, Case Western Reserve University School of Medicine, Cleveland, OH, USA, GenBank #AF004394) with BamHI and EcoRI and replacing X4 *env* with R5 *env* fragments.

### Luc assay

1G5 or 1G5/CCR5 cells were infected with 50 ng of p24-measured amounts of HIV-1_NL432_, HIV-1_NL-E_, HIV-1_NL-D_, or HIV-1_NLAD8-D_ per 1×10^5^ cells for 2 h, washing three times, and then culturied. After 48 h post-infection, cell lysates were prepared and the Luc assay was performed according to the manufacturer's instructions (Promega).

### Preparation of HIV-1 virus stocks

To prepare HIV-1 virus stocks, human embryonic kidney cell line 293T cells were transfected with 20 µg of pNL432, pNL-E, pNL-D or pNLAD8-D using the calcium phosphate precipitation method and then incubation for 48 h. Culture supernatant was treated with benzonase (1 U/ml) for 30 min at 37°C, cleared by filtration, and then frozen at −80°C. The amount of virus in each culture supernatant was measured by in-house HIV-1 Gag p24 ELISA [17].

### Cell culture

The 293T cells were maintained in Dulbecco's Modified Eagle Medium (Invitrogen [GIBCO], Carlsbad, CA, USA), supplemented with 10% heat-inactivated fetal bovine serum (FBS), penicillin (100 µg/ml), and streptomycin (100 µg/ml). 1G5 (obtained from AIDS Research and Reference Reagent Program, USA) and 1G5/CCR5 cells (CCR5 transfectants of 1G5 cells) were maintained in RPMI-1640 medium supplemented with 10% FBS, penicillin (100 µg/ml), streptomycin (100 µg/ml) (10% FBS-RPMI) and puromycin (2 µg/ml). CEMx174 CCR5/LTR-EGFP cells were maintained in RPMI-1640 medium supplemented with 10% FBS, penicillin (100 µg/ml), streptomycin (100 µg/ml) (10% FBS-RPMI), puromycin (2 µg/ml), and blasticidin (5 µg/ml) [36].

MDDC and T cells were prepared as described previously [46]. Blood samples were collected from healthy donors after we received written informed consent, and the collection was approved by the institutional ethical committee. The PBMCs were separated by a ficoll-hypaque density gradient (Lymphosepal: IBL, Gunma, Japan), enriched for CD14^+^ cells with magnetic anti-CD14 beads and a magnetic cell sorter (MACS, Miltenyi Biotec, Cologne, Germany), and cultured for 7 d in the presence of 10 ng/ml IL-4 and 10 ng/ml GM-CSF (both from PeproTech London, United Kingdom). CD4+ T cells were negatively selected by depletion using the EasySep human CD4^+^ T cell enrichment kit (StemCell Technologies, Vancouver, Canada). The purity of CD4^+^ T cells was >98%, as assessed by FACScalibur (BD Biosciences, San Jose, CA, USA).

### Kinetics of virus production in PHA blasts

PHA blasts were prepared by stimulating PBMCs with 5 µg/ml PHA and, 3 d later, infecting them with 20 ng of p24-measured amounts of HIV-1_NL432_, HIV-1_NL-E_, HIV-1_NL-D_, or HIV-1_NLAD8-D_ per 1×10^6^ cells for 2 h, washing them three times, and then culturing them with 10% FBS-RPMI containing a recombinant interleukin-2 (IL-2) 50 U/ml. Culture supernatants were harvested at 3–4 d intervals and viral production was monitored by in-house HIV-1 p24 Gag antigen ELISA.

For flow cytometric analysis of fluorescent proteins, HIV-1-infected PHA blasts were stained with APC-labeled anti-CD3 mAb for 15 min on ice, washed once, and resuspended in staining buffer (PBS, 2% FBS, and 0.05% sodium azide) containing 1 µg/ml propidium iodide (PI). These cells were analyzed by FACScalibur using the Cell Quest program. The FACS data were reanalyzed using Flowjo software by gating live (PI-negative) CD3^+^ T cells (Tree Star, San Carlos, CA, USA).

### Quantitative PCR and RT-PCR analysis

HIV-1_NL-E_- or HIV_NLAD8-D_-infected MDDCs or CD4^+^ T cells were collected and total DNA was prepared at 6 and 18 hpi. For the detection and quantification of individual forms of HIV-1 DNA, oligonucleotide primers and probe sequences were specifically designed for the TaqMan assay as described elsewhere [36]. All probes (Biosearch Technologies, Novato, CA, USA) were 5′ labeled with the fluorophore FAM as the reporter dye, and 3′ labeled with Black Hole Quencher-1 (BHQ-1) as the quencher dye. The amount of HIV-1–specific DNA per cell was normalized to that of the β-globin gene.

For allogeneic stimulation, primary CD4^+^ T cells (1×10^6^) were cocultured with allogeneic MDDCs (1×10^5^) in 96-well round bottom plates for 2 or 24 h. The cells were then washed with PBS, and anti-CD11c mAb was added to half of the cells to deplete MDDCs from the DC–T cell coculture. After incubating for 15 min on ice, the anti-CD11c-reacted cells were incubated with Dynabead M450 goat anti-mouse IgG (Dynal Biotech, Lake Success, NY, USA) for 30 min at 4°C, and then CD11c^+^ MDDCs were removed using a magnet stand. For TcR stimulation, primary CD4^+^ T cells were left unstimulated, weakly stimulated with 0.5 µg/ml anti-CD3 and 1 µg/ml anti-CD28, or strongly stimulated with 5 µg/ml anti-CD3 and 10 µg/ml anti-CD28 for 2 h. Total RNA was extracted from these CD4^+^ T cells, and qRT-PCR analysis was performed to measure the level of IFN-γ mRNA expression using the SuperScript III Platinum SYBR Green One-Step Quantitative RT-PCR system (Invitrogen). The sequences of the qRT-PCR primers were as follows: IFN-γ forward, 5′-tcccatgggttgtgtgttta-3′ and IFN-γ reverse 5′-aagcaccaggcatgaaatct-3′. The amount of IFN-γ mRNA was normalized to elongation factor 1 alpha (*EF-1*α) mRNA expression. The reaction was performed using an Mx3000P (Stratagene, La Jolla, CA, USA).

### HIV-1 infection of MDDCs and transmission to CD4^+^ T cells

MDDCs were left uninfected or were infected with 200 ng each of HIV-1_NL-E_ and HIV-1_NLAD8-D_ per 1×10^6^ cells for 2 h, washed three times, and then cultivated for 24 h in 24-well culture plates. HIV-infected or mock-infected MDDCs were collected, washed with PBS, treated with 0.025% trypsin for 5 min at 37°C, and then washed twice with 10% FBS-RPMI. MDDCs (0.5×10^5^ per well) were cocultured with autologous or allogeneic CD4^+^ T cells (0.5×10^6^ per well) in 96-well round-bottom plates. In some cases, purified protein derivatives of 25 µg/ml *Mycobacterium tuberculosis* (PPD) or a 10% final volume of CMV antigen (CMV_AD169_-infected MRC-5 lysate, kindly provided by N. Inoue, The first department of Virology, National Institute of Infectious Diseases, Tokyo, Japan ) were added to the culture. The CD4^+^ T cells stimulated with the weak or strong IL-2 protocol, described above, were left uninfected or were infected with 200 ng each of HIV-1_NL-E_ and HIV-1_NLAD8-D_ per 1×10^6^ cells for 2 h, extensively washed, and cultivated in 48-well tissue culture plates (1×10^6^ per well) in 10% FBS-RPMI containing IL-2. These culture supernatants were harvested at 3–4 d intervals, and viral production was monitored by p24 antigen ELISA.

For flow cytometric detection of R5 (DsRed^+^) and/or X4 (EGFP^+^) HIV-1-infected CD4^+^ T cells, cells were stained with APC-labeled anti-CD3 mAbs, and PI^−^ CD3^+^ cells were analyzed by FACScalibur using the Cell Quest program and reanalyzed using Flowjo software.
